# Supplementary material for: Hybrid Models and Biological Model Reduction with PyDSTool
Source: PLoS Comput Biol. 2012 Aug 9;8(8):e1002628. doi: 10.1371/journal.pcbi.1002628 (PMC3415397; doi:10.1371/journal.pcbi.1002628)
Supplement: Text S4 — Complete source code for the PyDSTool package (version 0.88.120504). Includes API documentation and help files linking to web pages. This file is identical to the current public release on Sourceforge.net. (ZIP) [file pcbi.1002628.s004.zip › PyDSTool/html/PyDSTool.PyCont.TestFunc.Hopf_Double_Bor_One-class.html]

xml version="1.0" encoding="ascii"?


PyDSTool.PyCont.TestFunc.Hopf\_Double\_Bor\_One


| Home | Trees | Indices | Help | PyDSTool |
| --- | --- | --- | --- | --- |

Package PyDSTool ::
Package PyCont ::
Module TestFunc ::
Class Hopf\_Double\_Bor\_One

[hide private]

[frames] | no frames]

# Class Hopf\_Double\_Bor\_One

source code

```
object --+            
         |            
  Function --+        
             |        
      TestFunc --+    
                 |    
      BorderMethod --+
                     |
object --+           |
         |           |
  Function --+       |
             |       |
      TestFunc --+   |
                 |   |
       BiAltMethod --+
                     |
                    Hopf_Double_Bor_One
```

---


|  |  |
| --- | --- |
| Instance Methods | [hide private] |

|  |  |
| --- | --- |
| \_\_init\_\_(self, F, C, update=False, save=False, numpoints=None)  F: R^a --> R^b | source code |

|  |  |
| --- | --- |
| setdata(self, X, V)  Note: p, q <= min(n,m) | source code |

|  |  |
| --- | --- |
| updatedata(self, A) | source code |

|  |  |
| --- | --- |
| func(self, X, V) | source code |

**Inherited from `BorderMethod`**:
`getVW`

**Inherited from `BiAltMethod`**:
`bialtprod`,
`bialtprodeye`

**Inherited from `TestFunc`**:
`diff`,
`findzero`

**Inherited from `Function`**:
`__call__`,
`__getitem__`,
`__setitem__`,
`numhess`

**Inherited from `object`**:
`__delattr__`,
`__getattribute__`,
`__hash__`,
`__new__`,
`__reduce__`,
`__reduce_ex__`,
`__repr__`,
`__setattr__`,
`__str__`


|  |  |
| --- | --- |
| Properties | [hide private] |

**Inherited from `object`**:
`__class__`


|  |  |
| --- | --- |
| Method Details | [hide private] |

|  |  |
| --- | --- |
| \_\_init\_\_(self, F, C, update=False, save=False, numpoints=None)  *(Constructor)* | source code |

F: R^a --> R^b

Note: I did not assign b automatically (although I could - it would
just be b = p\*q) since you may not want to use all of the entries of S.
Some bordering methods are not minimally augmented systems and thus only
require certain entries of S.

Overrides: object.\_\_init\_\_: *(inherited documentation)*

|  |  |
| --- | --- |
| setdata(self, X, V) | source code |

Note: p, q <= min(n,m)

Overrides: BorderMethod.setdata: *(inherited documentation)*

|  |  |
| --- | --- |
| updatedata(self, A) | source code |

Overrides: BorderMethod.updatedata

|  |  |
| --- | --- |
| func(self, X, V) | source code |

Overrides: BorderMethod.func

  


| Home | Trees | Indices | Help | PyDSTool |
| --- | --- | --- | --- | --- |

|  |  |
| --- | --- |
| Generated by Epydoc 3.0.1 on Fri May 4 15:24:07 2012 | http://epydoc.sourceforge.net |
